# Histaminergic system in brain disorders: lessons from the translational approach and future perspectives

**DOI:** 10.1186/s12991-014-0034-y

**Published:** 2014-11-18

**Authors:** Diego Baronio, Taylor Gonchoroski, Kamila Castro, Geancarlo Zanatta, Carmem Gottfried, Rudimar Riesgo

**Affiliations:** Translational Research Group in Autism Spectrum Disorders (GETTEA), Ramiro Barcelos, 2350 - Santa Cecília, Porto Alegre, RS 90035-903 Brazil; Postgraduate Program in Child and Adolescent Health, Federal University of Rio Grande do Sul, Porto Alegre, RS Brazil; Research Group in Neuroglial Plasticity, Department of Biochemistry, Federal University of Rio Grande do Sul, Porto Alegre, RS Brazil; Child Neurology Unit, Clinical Hospital of Porto Alegre, Federal University of Rio Grande do Sul, Porto Alegre, RS Brazil

**Keywords:** Antagonist, Autism, Brain disorders, Histamine, Histaminergic system

## Abstract

Histamine and its receptors were first described as part of immune and gastrointestinal systems, but their presence in the central nervous system and importance in behavior are gaining more attention. The histaminergic system modulates different processes including wakefulness, feeding, and learning and memory consolidation. Histamine receptors (H1R, H2R, H3R, and H4R) belong to the rhodopsin-like family of G protein-coupled receptors, present constitutive activity, and are subjected to inverse agonist action. The involvement of the histaminergic system in brain disorders, such as Alzheimer’s disease, schizophrenia, sleep disorders, drug dependence, and Parkinson’s disease, is largely studied. Data obtained from preclinical studies point antagonists of histamine receptors as promising alternatives to treat brain disorders. Thus, clinical trials are currently ongoing to assess the effects of these drugs on humans. This review summarizes the role of histaminergic system in brain disorders, as well as the effects of different histamine antagonists on animal models and humans.

## Introduction

Described for the first time in 1910 as a promoter of contraction of smooth muscles and vasodilatation, histamine acts as a transmitter in the central nervous system (CNS) and modulates several other physiological processes, like gastrointestinal and circulatory functions, innate and acquired immunity, cell proliferation and hematopoiesis. Today, its presence in the CNS and importance in behavior are largely studied [1,2].

Histamine synthesis and release are regulated by H3R, an autoreceptor present in the somata and axon terminals of histaminergic neurons. Histamine is synthesized from L-histidine by histidine decarboxylase, and it is metabolized by diamine oxidase and histamine *N*-methyltransferase (HNMT) [3]. Other receptors, such as muscarinic, opioid, and galanin, regulate histamine release in specific brain regions [4-6].

Histaminergic neurons are located in the tuberomamillary nucleus (TMN) of the hypothalamus, with widespread projections innervating most brain areas. Postmortem studies indicate that the number of histaminergic neurons in humans is about 64,000 [7]. There are four histamine receptors, all part of the rhodopsin-like family of G protein-coupled receptors (GPCR). Through these receptors, histamine regulates several basic body functions, such as wakefulness, feeding, and learning and memory [8-10].

## Histamine receptors

In 1966, Ash and Schild discovered the H1R while studying the effect of antihistamine drugs in the rat uterus and stomach [11]. After that, three other receptors (H2R, H3R, and H4R) were identified. The four receptors are part of the GPCR superfamily, and they all present constitutive activity [12-15].

The GPCR superfamily modulates several physiological processes and is divided into families and subfamilies, and single subtypes can present different isoforms. The discovery of constitutively active mutant receptors proved that these receptors could be activated without the presence of an agonist [16]. Binding of a ligand to a receptor may initiate activity (agonist with positive intrinsic activity) or prevent the effect of an agonist (antagonist with zero intrinsic activity). While the agonists stabilize the receptor in active conformation, the inverse agonists stabilize the receptor in inactive conformation and thus reduce the activity (negative intrinsic activity) [17]. Two isoforms of the H3R are highly constitutively active: the wild type and an isoform with a deletion in the third intracellular loop [18].

From the four histaminergic receptors, H1R is the main target of most of the approved drugs [19] and is found in different tissues and cells, including the smooth muscle, brain, and lymphocytes [20,21]. This receptor is the only member of the family of histamine receptors, of which a co-crystal was obtained, by using the first-generation antagonist doxepin (PDB ID 3RZE) [22]. As for all members of this family, this receptor comprises seven transmembrane helices (TMH), three intracellular loops, and three extracellular loops. The binding pocket of H1R has a conserved hydrophobic nature, which contributes to the low selectivity of doxepin and the first-generation of H1R antagonists, and is associated to an anion-binding region which has been related to the binding of second-generation H1R antagonists. Such crystallographic structure can be used as a model in further development of new blockers as well as in the understanding of the activation-inactivation mechanisms of this receptor family at molecular level. The signal transduction of H1R includes activation of phospholipase C, which promotes the inositol triphosphate-dependent release of Ca^2+^ from intracellular stores and diacylglycerol-sensitive activation of protein kinase C [2,23].

H1R is involved in the modulation of important processes and mice lacking this receptor present different impairments, for example, in spatial memory and in sleep-wake characteristics [24,25]. Injection of a H1R agonist in the median preoptic nucleus, a region involved in basal thermoregulation, induces persistent hyperthermia [26]. Other study with mutant mice lacking H1R suggested a role for this receptor in somatic and visceral pain perceptions. These animals showed fewer nociceptive responses to the hot-plate, tail-flick, tail-pressure, paw-withdrawal, formalin, capsaicin, and abdominal constriction tests [27].

The pioneer study by Ash and Schild indicated, at that time, the existence of at least two classes of histamine receptors, since the antagonists utilized in that experiment did not stop gastric acid secretion [11]. Later, compounds that blocked the gastric acid secretion in guinea pig, named H2R antagonists, were developed by Black and colleagues [28]. Like H1R, H2R presents typical GPCR receptor characteristics. Its activation stimulates adenylyl cyclase leading to cyclic adenosine monophosphate (cAMP) production, a second messenger that has different roles in the cell [29]. The role of H2R in the CNS is not fully understood, but basic research showed that it is related to the processes of learning and memory, motor control, and thermoregulation [30,31]. In some areas of the brain, colocalization of H1R and H2R suggests synergistic interactions between these two receptors [32]. In an animal model of multiple sclerosis, it was demonstrated that both H1R and H2R are propathogenic, mediating immune deviation and blood brain barrier (BBB) disruption [33].

Mainly found in the brain, H3R regulates food intake, memory, acetylcholine (ACh) release, and consolidation of fear memories [34,35]. Activation of H3R inhibits cAMP synthesis and activates MAP kinases and the AKT/GSK3β axis [36-38]. When activated, the receptor inhibits cell firing and decreases the release of histamine by histaminergic neurons, as well as inhibits secretion of norepinephrine, serotonin, and other neurotransmitters [13,39,40]. Recently, several alterations were reported in mice lacking H3R. They presented enhanced histaminergic neurotransmission, which led to changes in the mice phenotype, indicating a possible metabolic disorder as a consequence. The sleep was also altered, in a condition similar to sleep restriction in humans, which matches with obesity tendency presented by these animals [41].

Almost 15 years ago, H4R was the last histamine receptor to be identified. It is mainly related with immune functions, but its presence in the brain is known, as well as in the bone marrow, peripheral blood, spleen, thymus, small intestine, colon, heart, and lung [42-44]. It modulates different processes such as dendritic cells activity, interleukins release, and likely regulation of BBB permeability [45-47].

In humans, the presence of H4R messenger RNA (mRNA) was detected in the spinal cord, hippocampus, cerebral cortex, thalamus, and amygdala, with levels in the spinal cord overcoming the levels found in the spleen and liver. The authors also verified the presence of the receptor in the dorsal root ganglia, which might indicate a nociceptive role for the receptor. In rats, the cerebellum and hypothalamus presented the highest amounts of H4R mRNA [48].

## Histaminergic system and brain disorders

Alterations in the histaminergic system have been reported in several brain disorders and might have a significant role in their pathophysiology [49-52]. Considering this, it is not surprising that pharmacological studies are in development to explore the potentialities of histamine antagonists/inverse agonists in the treatment of these disorders. Table 1 shows the uses and outcomes of histamine receptors antagonists cited in this review.

**Table 1:** **Histamine receptors antagonists: therapeutic applications and outcomes**

**Receptor**	**Drug**	**Disorder**	**Study**	**Outcome**	**Reference**
H1R	Chlorpheniramine	Model of stress by immobilization/sleep disturb	Preclinical	Reduction in REM sleep.	[53]
	Dimebon	AD	Clinical	No significant improvement in a phase III trial.	[54]
	Doxepin	Insomnia	Clinical	Improvements in sleep maintenance and duration in a 4-week outpatient trial of elderly adults.	[55,56]
H2R	Dimebon	AD	Clinical	No significant improvement in a phase III trial.	[54]
	Famotidine	Autism	Clinical	Attenuated symptoms like irritability, hyperactivity and atypical pattern of eye contact in children with autism.	[57]
		SCH	Clinical	Reduced scores in BPRS, CGI, and SANS.	[58,59]
H3R	GSK239512	AD	Clinical	Failed on improving executive function/working memory in a randomized, double-blind, placebo-controlled.	[60]
	ABT-288	AD	Clinical	No significant improvements in a randomized study.	[61]
		SCH	Clinical	Failed on providing cognitive improvements to patients.	[62]
	JNJ-10181457	Model of AD	Preclinical	Reversed cognitive deficits induced by scopolamine and normalized ACh neurotransmission.	[63]
	Pitolisant	Narcolepsy	Clinical	Reduced excessive daytime sleepiness	[64]
	ABT-239	Model of SCH	Preclinical	Attenuated cognitive deficits caused by ketamine and MK-801.	[50]
	A-431404	Model of SCH	Preclinical	Attenuated cognitive deficits caused by ketamine and MK-801.	[50]
	JNJ-31001074	ADHD	Clinical	No significant improvements in adult patients.	[65]
	Betahistine	SCH	Clinical	Reduced weight gain by patients with SCH treated with olanzapine.	[66]
	JNJ-39220675	Model of alcoholism	Preclinical	Reduced intake of alcohol after a period of abstinence.	[67]
	Thioperamide	Model of SCH	Preclinical	Enhancement of prepulse inhibition.	[68]
		Model of PD	Preclinical	Decreased hyperactivity.	[69]
	GSK189254	Model of neuropathic pain	Preclinical	Antinociceptive effect.	[70,71]
	GSK334429	Model of neuropathic pain	Preclinical	Antinociceptive effect.	[71]
	SAR110894	Model of SCH	Preclinical	Normalized impaired social behavior.	[72]
	Ciproxifan	Model of SCH	Preclinical	Enhancement of prepulse inhibition.	[68]
		Model of AD	Preclinical	Improvements in hyperactivity and memory deficits.	[73]
H4R	JNJ7777120	Model of neuropathic pain	Preclinical	Antinociceptive effect.	[74,75]
	ZPL3893787	Tested in healthy volunteers. Potential treatment for asthma, allergic rhinitis, pain, and other inflammatory diseases.	Clinical	Completed phase I trial. Safe and well tolerated.	[76]
	UR63325	Allergic rhinitis	Clinical	Currently on phase II clinical trial. No data available.	[76]
	KD1157	Tested in healthy volunteers. Potential treatment for allergic rhinitis.	Clinical	Safe and well tolerated.	[76]
	JNJ38518168	Asthma	Clinical	Currently on phase II clinical trial. No data available.	[76]
	JNJ39758979	Asthma	Clinical	Completed phase II clinical trial. No data available.	[76]

### Alzheimer’s disease

Alzheimer’s disease (AD) affects approximately 5.4 million US citizens and the costs with patients, including health care, long-term care, and hospitalization, make it a social problem [77]. Even though there is conflicting information about the role of histaminergic system in AD, abnormalities have been reported. A postmortem study showed reduced histamine content in the hypothalamus, hippocampus, and temporal cortex of patients with AD [7]. Other report showed 57% less histaminergic neurons in the TMN of AD patients and females had increased prefrontal cortex expression of H3R. Despite the severe cell loss, it is suggested that histamine production is not affected, since the levels of histidine decarboxylase mRNA are unaltered [78]_._ On the other hand, Kim and colleagues reported decreased levels of the histamine-releasing factor in the temporal cortex of patients with AD, as well as in the temporal cortex, thalamus, and caudate nucleus of patients with Down syndrome, indicating low levels of brain histamine in these disorders [79].

The development of new treatments for AD is a permanent issue. Positive results after a double-blind, placebo-controlled, phase II trial led to great expectations about the possibility of dimebon becoming a drug to treat AD, with patients displaying significant improvement over baseline for Alzheimer’s disease Assessment Scale [80]. Dimebon acts as an antagonist of H1R and H2R, and it also binds to adrenergic, serotonergic, and dopaminergic receptors. Unfortunately, a phase III trial with 598 patients found no significant improvement in patients with AD treated with this drug [54].

In a preliminary investigation GSK239512, an H3R antagonist/inverse agonist was tested in order to assess its tolerability and cognitive effects in patients with AD. The patients displayed no abnormalities in hematology, clinical chemistry, urinalysis parameters, and cardiovascular parameters. Enhancements in cognition were assessed, with subjects showing positive effects on attention and memory [60]. In a larger trial, GSK239512 was considered safe and able to improve episodic memory in patients with AD, but it failed on improving executive function/working memory [81]. Other H3R antagonists, such as ABT-288, have also failed in clinical trials [61].

On the other hand, interesting findings were reported when ciproxifan, also H3R antagonist, was tested in a transgenic mouse model of AD. Improvements in some impairments featured in this disorder, such as hyperactivity and memory deficits were detected after administration of this drug [73]. This was also performed with rats that received scopolamine, a non-selective muscarinic antagonist that induces cognitive impairments and reduces ACh release, similar to what is found in AD. Treatment with JNJ-10181457, a selective non-imidazole H3R antagonist, not only reversed cognitive deficits induced by scopolamine but also normalized ACh neurotransmission [63].

### Sleep disturbs

Histamine has an important role in the control of sleep-wake regulation. Studying histaminergic TMN neurons of cats in different states, Vanni-Mercier reported a slow, but regular spontaneous firing during wake state, diminished in slow-wave sleep and absent firing during rapid eye movement [82]. Histidine decarboxylase gene knockout mice showed that impaired histamine synthesis prevents remaining in a wake state [83]. In agreement with that, patients with narcolepsy and idiopathic hypersomnia present reduced level of histamine in the cerebrospinal fluid (CSF) [84].

In rats, inverse agonism of H1R with ketotifen increased non-rapid eye movement (REM) sleep and decreased REM sleep, coinciding with significant prolongation of sleep duration and longer slow-wave sleep, suggesting a restful sleep [85]. In a model of stress by immobilization, the percentage for REM sleep is increased but is abolished in rats after administration of an H1R antagonist/inverse agonist, chlorpheniramine. The reduction of REM sleep caused by chlorpheniramine administration was significant even when compared to non-stressed control rats [53].

Recently, doxepin efficacy and tolerability were tested in elderly patients with chronic primary insomnia. During 4 weeks, 130 patients received 6 mg of doxepin nightly while 124 patients received placebo. According to patient self-report instruments and clinician ratings, the treatment was well tolerated and led to significant improvements in sleep maintenance and duration [55]. Another recent trial investigated the effects of doxepin in Parkinson’s disease (PD) patients with insomnia. Non-pharmacological treatment, 10 mg of doxepin nightly and placebo were compared during 6 weeks. Sleep variables were improved in the doxepin-treated and in the non-pharmacological groups, with the doxepin treatment appearing to have more substantial clinical benefits. The authors do not claim for a superiority of pharmacological treatment and point to advantages of the non-pharmacological, such as absence of pharmacological side effects and potential long-lasting benefits after treatment [56].

Beneficial effects of histamine antagonists are also seen in narcoleptic patients that received 40 mg of tripolisant daily for a week. The H3R inverse agonist was considered safe, and no serious adverse effects were reported during the study. Despite the small population studied, the authors highlight that after treatment, patients presented a reduction in somnolence, compared to baseline, equivalent to results after several months of modafinil treatment [64]. The same drug was tested in four teenagers with narcolepsy-catalepsy that presented severe daytime sleepiness and were refractory to available treatments. Pitolisant decreased sleepiness with few minor adverse effects [86]. In positron emission tomography (PET) study, after calculating H3R occupancy, it was verified that AZD5213 is able to exert its wake-promoting action during daytime and does not disrupt sleep during the night. This is consequence of its pharmacokinetics that allows circadian fluctuations of H3R occupancy. Thus, patients would benefit from the procognitive effects of H3R without presenting sleep disruption [87].

### Schizophrenia

Schizophrenia (SCH) is a heterogeneous disorder with strong genetic influence, highly prevalent, affecting 1.1% of the US population [88]. The involvement of the dopaminergic system in the pathophysiology of this disorder is well known, with antipsychotics acting mainly on D_2_ receptor [89]. However, a role for the histaminergic system has been proposed, and several novel pharmaceutical targets are acting on both dopaminergic and histaminergic systems [90]. Iwabuchi and colleagues evaluated the distribution of H1R in the brains of medicated schizophrenic patients and normal human subjects but found no discrepancy between the groups. They also calculated the binding potential of the receptor by PET and doxepin, a radioligand for H1R, and noted that the value of the binding potential was particularly lower in the frontal and prefrontal cortices and the cingulate gyrus of patients with SCH [51]. Analyzing postmortem brain samples of patients with SCH, Jin and colleagues found that medicated patients displayed lower H3R binding level in the hippocampal CA2 region. The prefrontal cortices of the same patients presented higher H3R radioligand binding than the controls’, and this alteration was correlated with psychotic symptoms, indicating a role of H3R in modulation of cognition [91]. Recently, it was reported that a H4R polymorphism may be a molecular marker for the prediction of risperidone efficacy [92].

In the 1990s, the effect of an H2R antagonist, commonly used in the treatment of peptic ulcer, was evaluated in patients with SCH. During 3 weeks, in an open-label trial, ten patients received 20 mg twice a day of famotidine without interrupting their treatment with conventional antipsychotics. Famotidine led to significant reduction in the scores of Brief Psychiatric Rating Scale (BPRS) and Clinical Global Impression (CGI), suggesting its administration as a useful alternative for SCH treatment [58]. Later, another open-label trial was performed with 18 patients receiving 100 mg of famotidine daily, during 3 weeks. Significant improvements were found after measurements with BPRS, CGI, and the Schedule for the Assessment of Negative Symptoms (SANS) [93]. Recently, a randomized clinical trial for famotidine was performed with 30 patients with SCH, 16 patients received 100 mg of famotidine twice daily and 14 received placebo. Famotidine caused no significant adverse effects, and it led to great reduction in symptoms for both the Positive and Negative Syndrome Scale and CGI [59].

In an animal model of SCH, impaired prepulse inhibition was enhanced in the DBA/2 mouse after inverse agonism of H3R with thioperamide and ciproxifan [68]. The use of ABT-239 and A-431404, also non-imidazole H3R antagonists, attenuated cognitive deficits caused by ketamine and MK-801 in rats, showing better results than antipsychotics, olanzapine, and risperidone, also used in the study, to treat these deficits [50]. Antagonists/inverse agonists of H3R have also shown to possess antioxidant activity, which could supplement antioxidant needs of SCH [94]. Despite the promising results obtained in preclinical models, when ABT-288 was tested in a randomized trial, it failed in providing cognitive improvements to patients with SCH. In addition, this treatment was associated with sleep disruption [62]. Other reports of disappointing results regarding the use of H3R antagonists in the treatment of disorders, such as attention-deficit hyperactivity disorder (ADHD), raise questions about the practicability of these drugs on the translational level [65].

Weight gain is a common side effect associated with some antipsychotic agents that may affect adherence to treatment [95]. Recently, the use of H3R antagonists was investigated as an alternative to attenuate weight gain caused by olanzapine in patients with SCH. A combination of reboxetine, a selective norepinephrine reuptake inhibitor, and betahistine, a potent antagonist of H3R was tested in a double-blind placebo-controlled study. The combination of 4 mg of reboxetine and 48 mg of betahistine was given daily, for 6 weeks, to 29 patients treated with olanzapine. Placebo was given to 14 patients also treated with olanzapine. The combination of reboxetine/betahistine resulted in significantly less weight gain compared to the placebo group [66].

### Addiction

It is known that alterations in different neurotransmission systems, such as glutamate system dysfunction, interaction between serotonin transporter and serotonin receptor 1B genes polymorphisms, and dopamine-beta hydroxylase polymorphism have been associated with drug dependence [96-98]. The histaminergic system might as well be involved in modulation of behaviors associated with addiction. A polymorphism of HNMT gene was found in abundance in alcoholics from two different populations. Higher levels of the enzyme HNMT may lead to decreased levels of histamine and the low levels of this amine could be linked to an anxious behavior, since the patients with the polymorphism also displayed higher harm avoidance, a dimensional rating of anxious personality. Vulnerability to alcohol dependence is commonly associated with anxious behavior. In animals, it was demonstrated that stimulation of H1R modulates an anxiogenic effect, while H2R does the opposite. The authors speculate that the carriers of the polymorphism may present low levels of histamine in the amygdaloid nuclei, a structure associated with anxiety and with high density of H2R [99].

Nuutinen and colleagues showed that when alcohol solution and plain water were offered to mice lacking H3R in a two-bottle choice procedure, these animals would drink less alcohol solution, compared to control animals. Also, in animals without the H3R, alcohol did not generate a rewarding effect, as well as not impaired motor coordination [100]. Similar results were obtained when rats were treated with JNJ-39220675. After 3 days of deprivation, rats were exposed to alcohol again 15 min after receiving a subcutaneous injection of JNJ-39220675. The animals displayed reduced intake of alcohol after a period of abstinence, just when the urge for drinking is enhanced [67]. Clinical studies should be performed to assess potential benefits and risks of these drugs to treat alcoholic patients.

### Parkinson’s disease

Reports of higher levels of histamine in the blood of patients with PD and in postmortem studies indicate a role for the histaminergic system on this disease [101]. The histaminergic innervation in the middle portion of substantia nigra pars compacta and reticulata was increased in the brains of patients with PD, which may be due to a compensatory event caused by deficiency of dopamine or a putative fiber growth inhibitory factor [102]. In postmortem brain samples of patients with PD, histamine concentrations were significantly increased in the putamen, substantia nigra pars compacta, internal globus pallidus, and external globus pallidus when compared to age-matched controls. This is probably a consequence of reduced metabolism, since the concentrations of tele-methylhistamine, a histamine metabolite, were unchanged [103]. Other study reported a significant decrease in the expression of H3R mRNA and a significant increase of HNMT mRNA expression, both in the substantia nigra of patients with PD. The disease duration (years between diagnosis and death) was negatively correlated with HNMT mRNA expression [104].

In a rat model of PD, obtained by brain lesion after bilateral *icv* administration of 6-hydroxydopamine, high levels of histamine were identified. Increased locomotor activity caused by the lesion and stereotyped behavior promoted by injection of apomorphine, were attenuated by administration of thioperamide, a H3R antagonist. Antagonists of H1R and H2R were also tested, but the symptoms caused by the lesion were not attenuated [69].

### Pain

The histaminergic system has a role in nociception, with histaminergic neurons projecting to the dorsal raphe nucleus and dorsal horn of the spinal cord. The H3R antagonists GSK189254 and GSK334429 demonstrated to be promising therapies for the treatment of neuropathic pain, since animals treated with these drugs presented a decrease in paw withdrawal threshold in the chronic constriction injury and varicella-zoster virus models [70]. In addition, GSK189254 produced antinociceptive effects in the model of monoiodoacetate induced osteoarthritic pain and in a spinal nerve ligation model of neuropathic pain [71].

Antinociceptive properties of H4R antagonists were also detected. JNJ7777120 was tested in four different strains of mice in order to verify its efficacy in a model of croton oil-induced ear inflammation. Reduction in ear edema and significant anti-inflammatory effects were detected in the animals treated with this drug [74]. In other study, after repeated administration, JNJ7777120 demonstrated an anti-hyperalgesic action in a model of neuropathic pain. It is not clear how this effect is produced, although JNJ7777120 possesses anti-inflammatory properties, it is also possible that a central effect is produced due to its capacity to cross the BBB [75]. Clinical studies are currently ongoing in order to evaluate the therapeutic potential of H4R antagonist in other inflammatory diseases, due to the involvement of H4R in immune regulatory functions, including chemotaxis and cytokine secretion [76].

## Perspectives

Over the last years, the role of histaminergic system has been studied in the pathophysiology of different brain disorders. Progress in this field of study has been made, making it possible to investigate different pharmacological approaches in order to treat or ameliorate symptoms. Autism, a neurodevelopmental disorder, affects 1 in 68 children in the US and has not a clear etiology or specific biomarkers [105]. Literature presents scarce data about the histaminergic system in autism (Table 2), but the use of an H2R antagonist has been already proposed and tested in patients. Symptoms like irritability, hyperactivity, and atypical pattern of eye contact were attenuated after treatment with famotidine [57,106].

**Table 2:** **Studies involving autism and histamine**

**Year**	**Author**	**Number of patients**	**Number of controls**	**Main results**
1972	Neville et al.	7	-	Elevated plasma histidine and low skin histidase levels [107].
1979	Kotsopoulos and Kutty	1	-	Patient with autism presented histidine blood levels seven times higher than the upper normal values [108].
1988	Launay et al.	22	22	Histamine levels in urine and whole blood or plasma of patients with autism did not differ from age- and sex-matched controls [109].
1999	Rossi et al.	25	-	Niaprazine (H1R antagonist) showed a positive effect on hyperkinesias, unstable attention, resistance to change and frustration, mild anxiety signs, hetero-aggressiveness, and sleep disorders [110].
2001	Linday et al.	9	-	Behavioral improvement in children treated with Famotidine (H2R antagonist) [57].
2010	Rosales-Reynoso et al.	10	10	Downregulation of H3R in patients with Fragile X syndrome, subjects that usually meet diagnostic criteria for autism [111].
2012	Ming et al.	48	53	Reduced urinary levels of histidine and other amino acids [112].
2013	Naushad et al.	138	138	When compared to normal controls, autistic children showed elevated levels of histidine (58 +/− 15 vs. 45 +/− 21 micromol/L) [113].

There is evidence that H3R is downregulated in Fragile X syndrome patients, a condition that is strongly associated with autism [111]. In addition, animals exposed to phencyclidine (PCP) develop behavioral impairments, including low interest in social novelty, which is a feature present also in autism. In this experiment, animals exposed to PCP spent 3.5 less time investigating a novel subject than the control group. The administration of a H3R antagonist/inverse agonist, SAR110894, normalized this impairment [72]. Recent data points to an involvement of the histaminergic system in the pathophysiology of Tourette’s syndrome, a condition common among patients with autism. A premature termination codon (W317X) in the histidine decarboxylase gene was detected in patients, implying that diminished histaminergic neurotransmission could be related to the outcomes of this syndrome [114].

Since it is likely that the histaminergic system may play a role in SCH and Tourette’s syndrome, disorders that have substantial symptomatic overlap with autism, we think that further investigation should be made to characterize this system in autism. The animal model of autism based on prenatal exposure to valproic acid shows neuroanatomical, behavioral, and biochemical alterations that recapitulates the core characteristics of autism [115], which makes it a reliable tool for studying a likely involvement of the histaminergic system in this disorder.

## Concluding remarks

Initially described as part of immune and gastrointestinal systems, the presence of histamine and its four described receptors in the CNS have been related to normal and/or abnormal behavior. As a result, a growing amount of information regarding the relationship between histamine and brain is continuously arising from both experimental and clinical fields of research.

Based on preclinical data, different antagonists from histamine receptors have been considered promising therapies for brain disorders. On the other hand, more clinical studies are still required to verify practicability of these drugs. We believe that in-depth investigation involving the histaminergic system and its potential therapeutic targets in other disorders, such as autism, should be performed. Efforts in both preclinical and clinical research will lead to reaching clinically useful and safe treatments.

## Abbreviations

H1R: histamine H1 receptor
H2R: histamine H2 receptor
H3R: histamine H3 receptor
H4R: histamine H4 receptor
CNS: central nervous system
HNMT: histamine *N*-methyltransferase
TMH: transmembrane helices
TMN: tuberomamillary nucleus
GPCR: G protein-coupled receptors
BBB: blood brain barrier
AD: Alzheimer’s disease
SCH: schizophrenia
PD: Parkinson’s disease
ACh: acetylcholine
PCP: phencyclidine
PET: positron emission tomography
BPRS: brief psychiatric rating scale
ADHD: attention-deficit hyperactivity disorder
CGI: Clinical Global Impression
